# Supplementary material for: Class I HLA Allele Predicted Restricted Antigenic Coverages for Spike and Nucleocapsid Proteins Are Associated With Deaths Related to COVID-19
Source: Front Immunol. 2020 Dec 16;11:565730. doi: 10.3389/fimmu.2020.565730 (PMC7772148; doi:10.3389/fimmu.2020.565730)
Supplement: Supplementary file 6 [file Presentation_1.pdf]

Supplementary Material

1 Supplementary Data

Supplementary_File_1. Epidemiological data.

Supplementary_File_2. HLA-I A and B allelic frequency distribution

Supplementary_File_3. Population coverage data generated from the IEDB

Supplementary_File_4. HLA Allelic frequency distribution for USA subpopulations

Supplementary_File_5 Candidate peptides for vaccination and HLA alleles associated with them

2 Supplementary Figures and Tables

2.1 Supplementary Figures

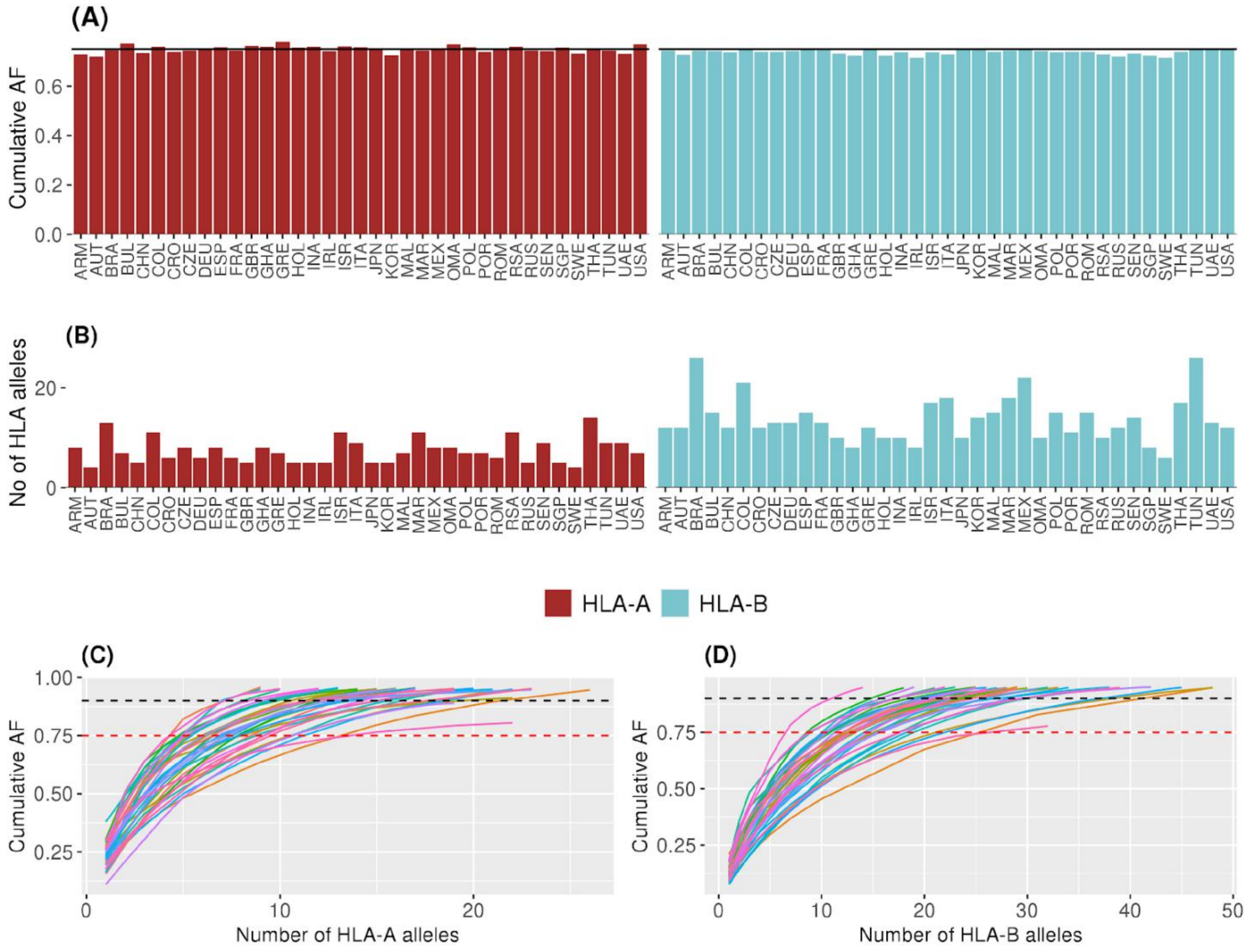

Supplementary Figure 1. HLA-I A and B allelic frequency coverage. **(A)** The allelic frequency coverage for each population (n=37) included in the study. Horizontal lines indicate the threshold (0.75). **(B)** The number of HLA-I A and B alleles to reach the allele frequency coverage threshold of 0.75 per studied population. **(C-D)** Rarefaction plots for HLA-A **(C)** and HLA-B **(D)** showing the approximation to a plateau with the increase of cumulative allele frequency (AF). Each color represents a different population in the study. Dashed lines represent cumulative AF of 0.75 (red) or 0.90 (black). For the rarefaction plots, up to 188 HLA alleles were used.

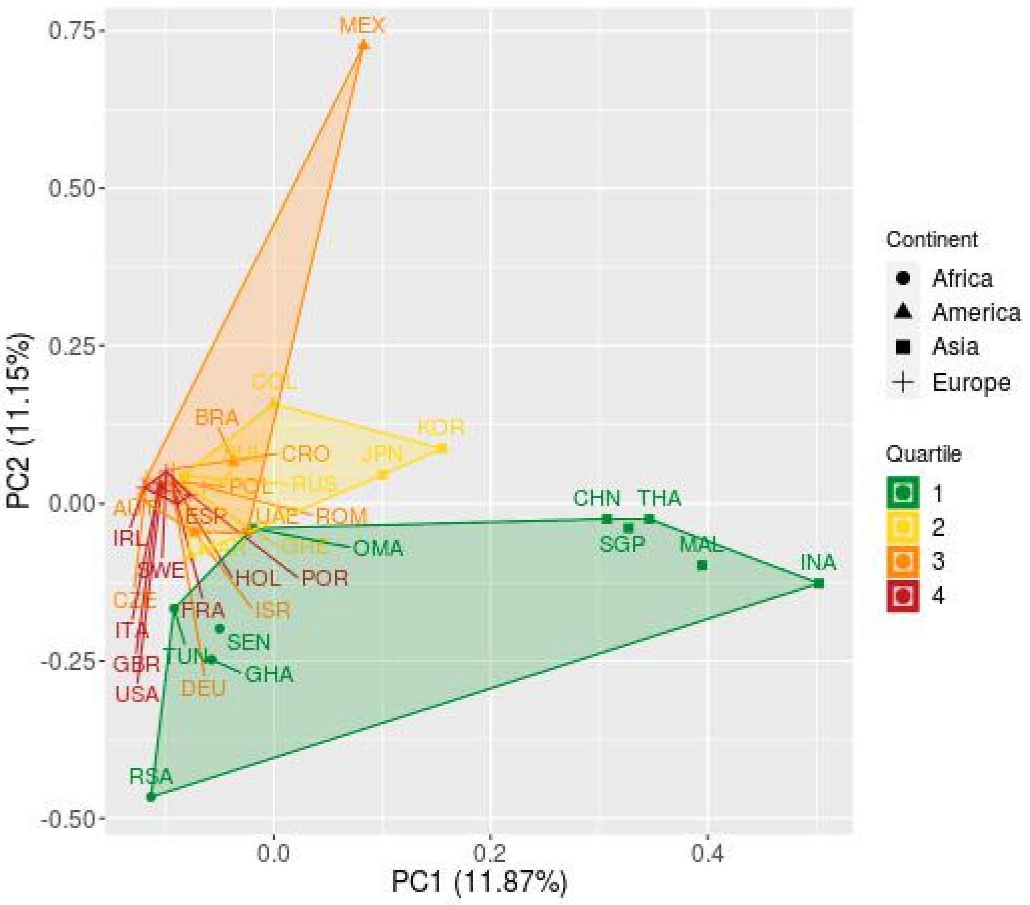

Supplementary Figure 2. Principal Component Analysis of 37 countries by HLA-A and HLA-B Allele frequencies. Geometric shapes represent continents, and colors represent deaths per million inhabitants quartiles: 1 = green (lower than 5); 2 = yellow (5 to 20); 3 = orange (21 to 85); 4 = red (greater than 85). Polygons were drawn to evidence countries belonging to the same quartile.

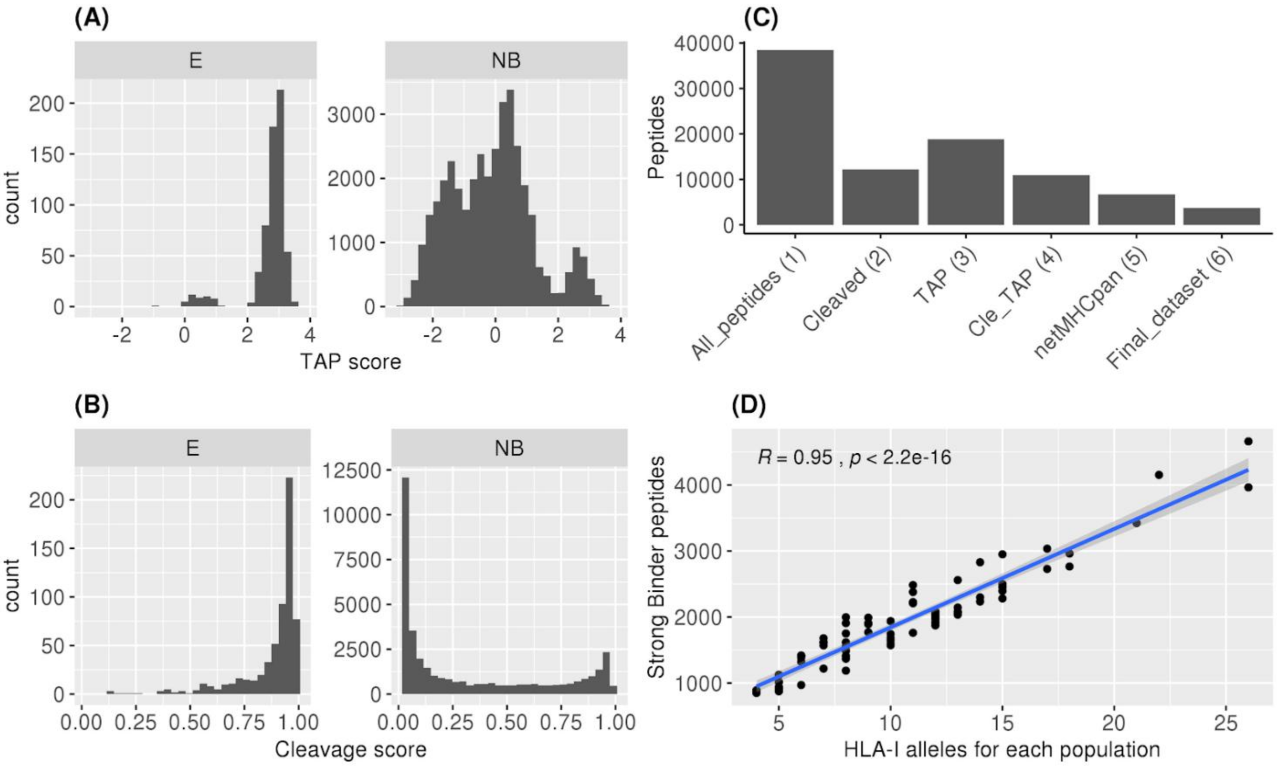

Supplementary Figure 3. (A) Distribution of TAP transport score according to predictions performed by netCTLpan. Left panel represents predicted epitopes (E) and the right panel non-binder peptides (NB). Peptides with a score below 0 were filtered out. **(B)** Distribution of proteasome cutting score performed as in **(A)**. Peptides with a score inferior to 0.5 were filtered out. **(C)** Summary of filtering steps evidencing the initial number of peptides (1), those that passed proteasome score (2), TAP transport (3), both proteasome and TAP processing (4), peptides predicted to bind HLA according to netMHCpan4 (5), and selected peptides consisting on the intersection between binding predictions by netMHCpan4 and proteasome and TAP processing (6). **(D)** Pearson's correlation between the number of non-unique Strong Binder peptides (Y-axis) and the number of HLA-I alleles for each population (X-axis). Each dot represents the number of HLA-I alleles selected for a given population.

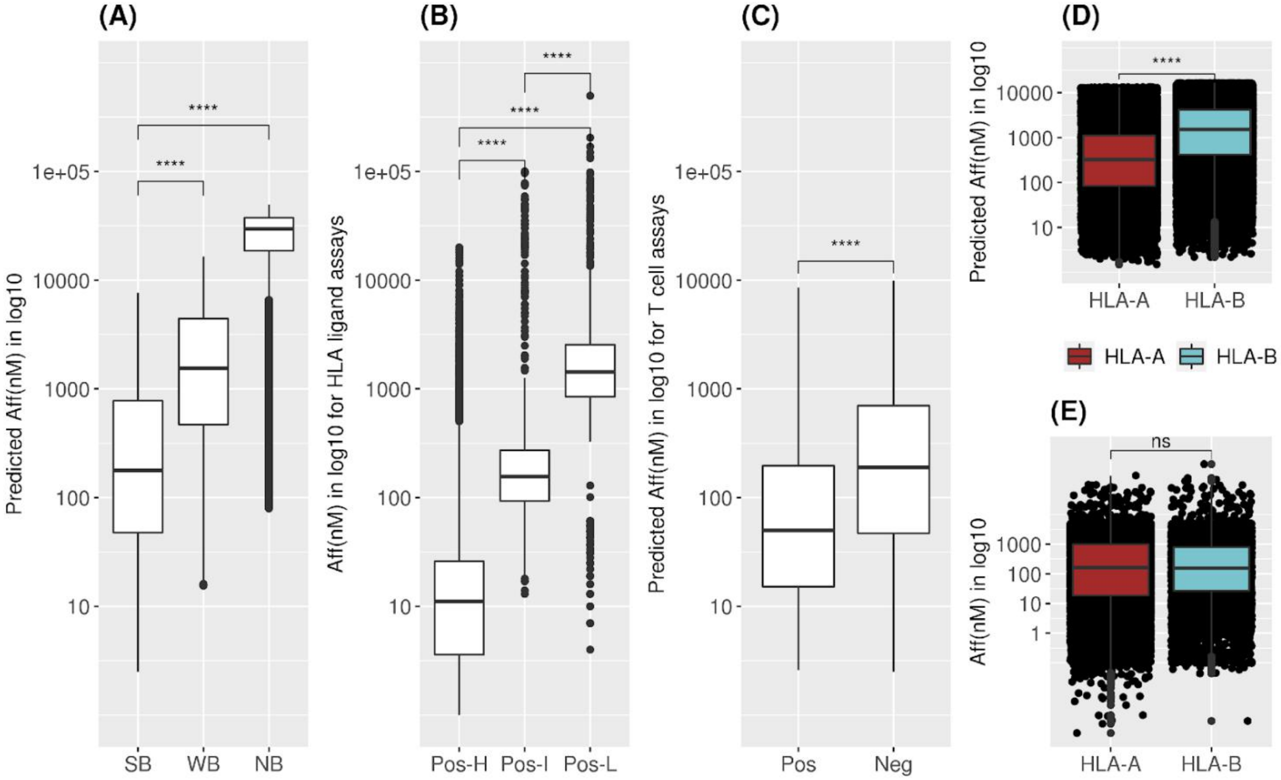

Supplementary Figure 4. Comparison between experimental binding affinities from Immune Epitope Database (IEDB) and *in silico* predictions. **(A)** Binding affinities (nM) predicted by netMHCpan for 8-11 mer peptides from SARS-CoV-2 and the 106 HLA-I alleles used in this analysis. **(B)** Experimental binding affinities (nM) for viral peptides and different HLA-I alleles according to the experimental read-out. Only linear virus-derived epitopes in HLA-I A and B with positive assays described were included. **(C)** Binding affinities predicted as in **(A)** for peptides that are capable (Pos) or not (Neg) to elicit immune response according to T cell assays. **(D-E)** Same data as in **(A)** and **(B)**, respectively, segregated by HLA class I gene. In **(D)** only SB and WB data were used. Pos, Positive; Pos-H, Positive-High; Pos-I, Positive-Intermediate; Pos-L, Positive-Low; Neg, Negative; SB, Strong Binder (%Rank <0.5); WB, Weak Binder (0.5≤ %Rank <2); NB, Non Binder (%Rank ≥2); ****p-value < 0.0001; n.s., not significant.

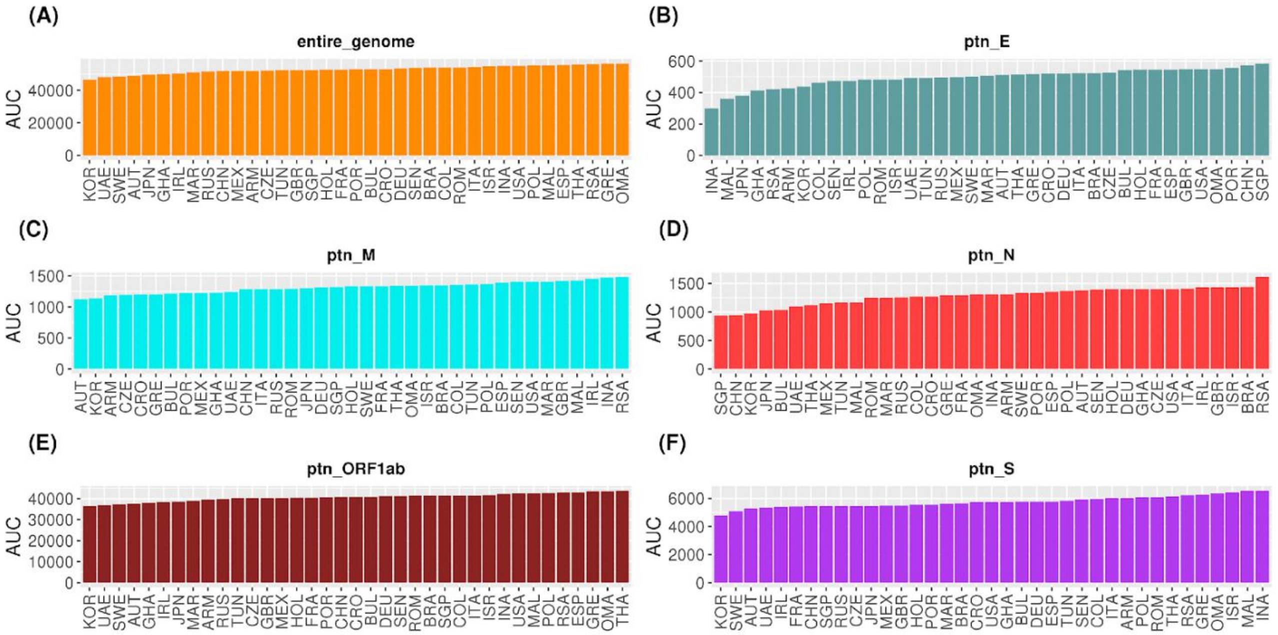

Supplementary Figure 5. Area Under the Curve (AUC) calculated for each population coverage curve shown in Figure 3. **(A)** AUC of the population coverage for the entire SARS-CoV-2 genome and **(B-F)** for selected proteins. The colors represent different proteins. E, Envelope; M, Membrane; N, Nucleocapsid; S, Spike protein.

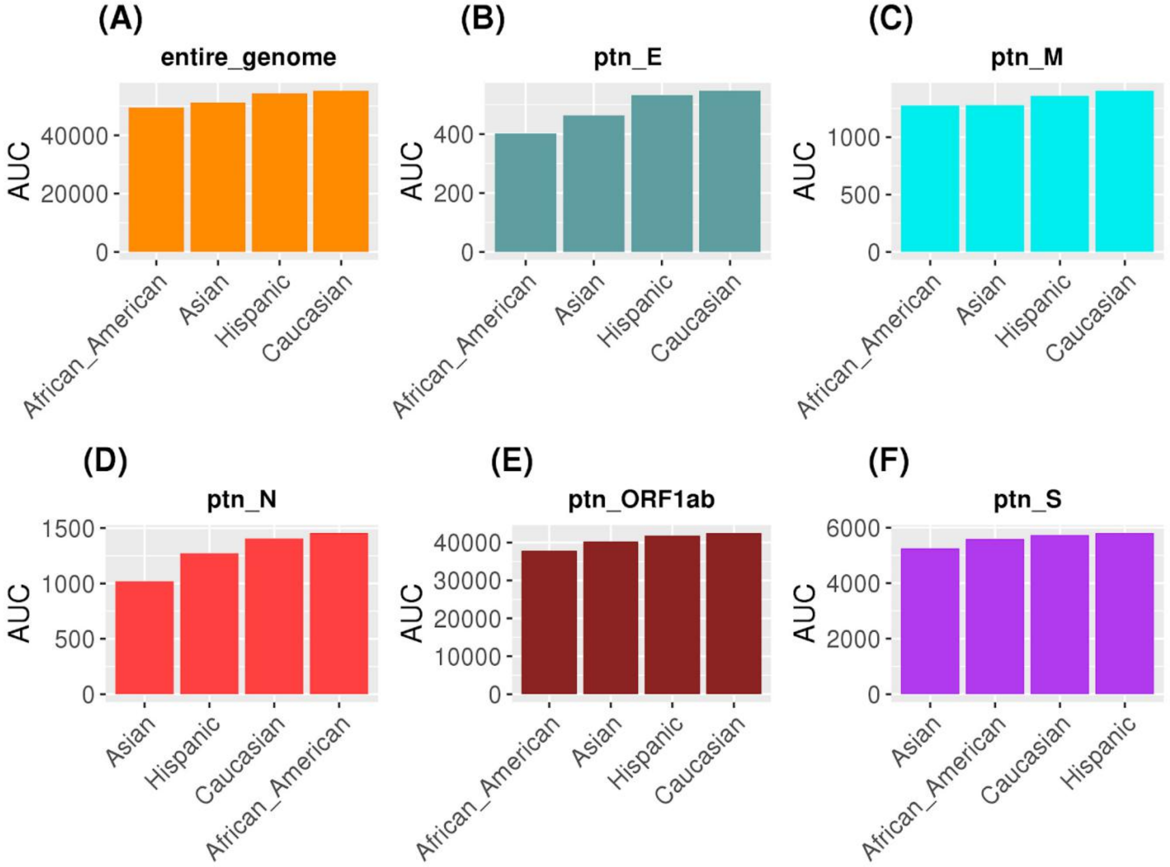

Supplementary Figure 6. Area Under the Curve (AUC) calculated for each population coverage curve considering 4 ethnic backgrounds in the USA population. **(A)** AUC of the population coverage for the entire SARS-CoV-2 genome and **(B-F)** for selected proteins. The colors represent different proteins. E, Envelope; M, Membrane; N, Nucleocapsid; S, Spike protein.

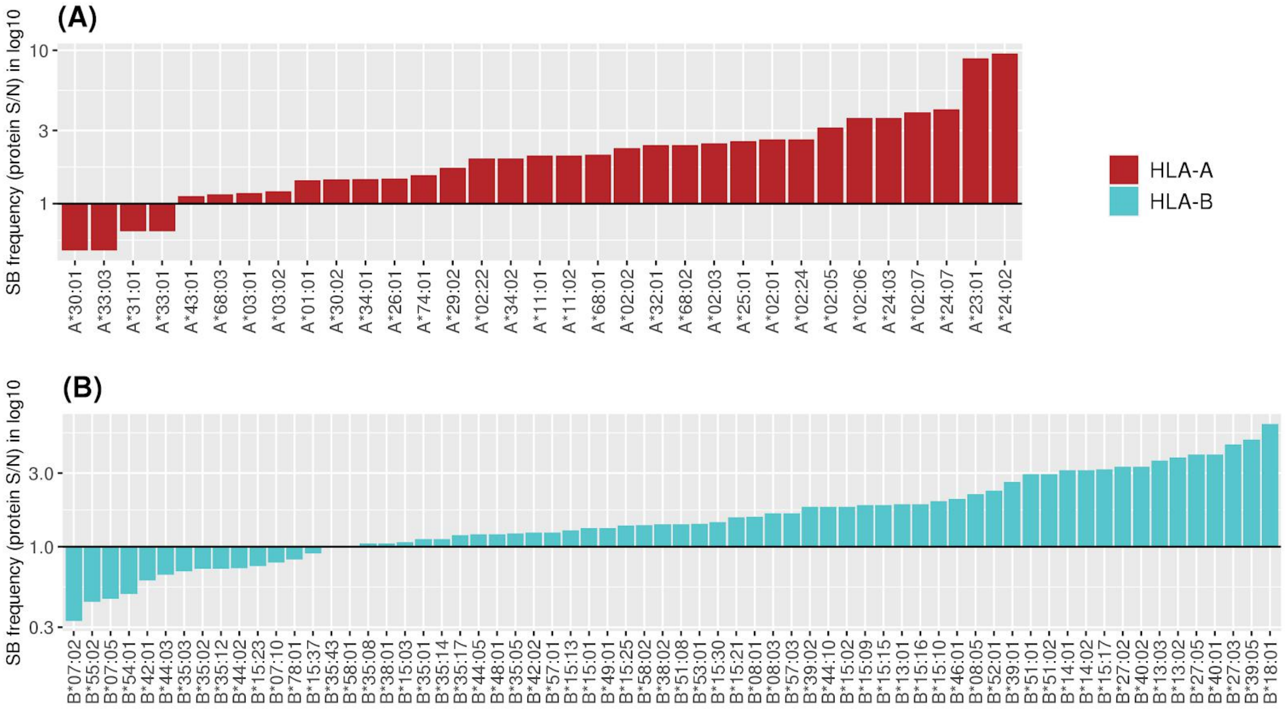

Supplementary Figure 7. Ratio between frequencies of SB peptides from protein S and N that are predicted to bind to HLA-A **(A)** or HLA-B **(B)**. The number of SB peptides was normalized dividing to the respective length in aa of each protein prior to the ratio calculation.

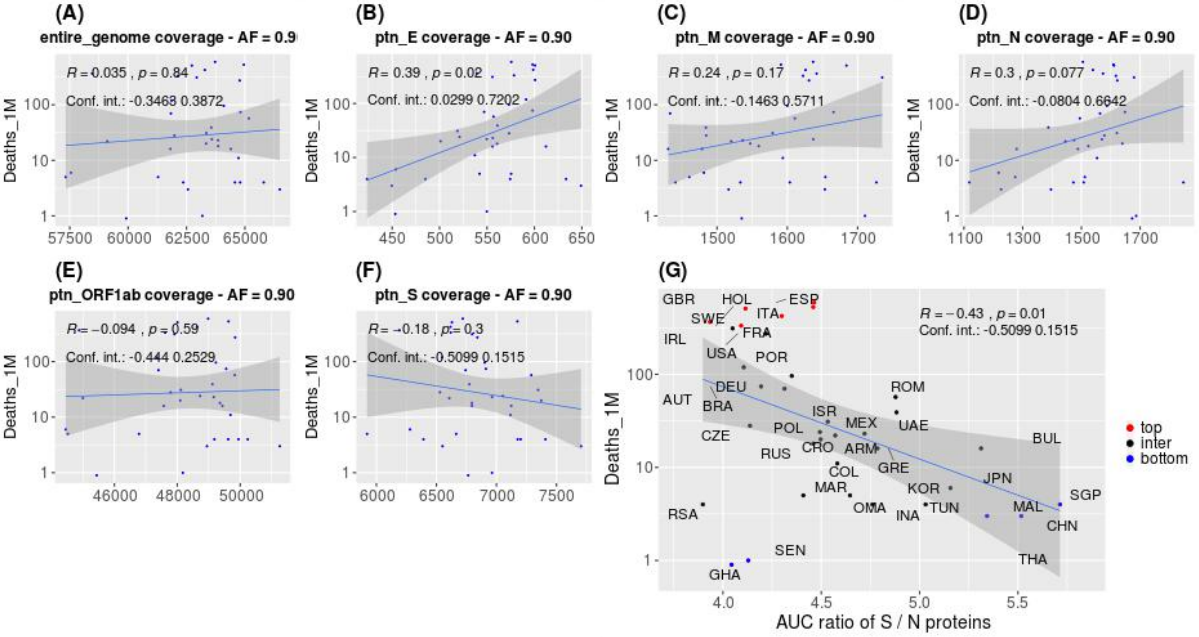

Supplementary Figure 8. Correlations between SARS-CoV-2 derived SB peptides coverage of each population and COVID-19 outcome. Spearman correlations between AUC calculated from the population coverage (cumulative AF: 0.90) considering the entire SARS-CoV-2 genome **(A)**, Envelope **(B)**, Membrane **(C)**, Nucleocapsid **(D)**, ORF1ab **(E)** or Spike protein **(F)** derived peptides and Deaths per million inhabitants. **(G)** Spearman correlation between the S/N AUC ratio and deaths per million inhabitants. Colors represent countries with a higher (red) or lower (blue) number of deaths. For this analysis, a threshold of 0.90 for the allele frequency was considered. The confidence interval shown was calculated using bootstrap with 1000 replacements.

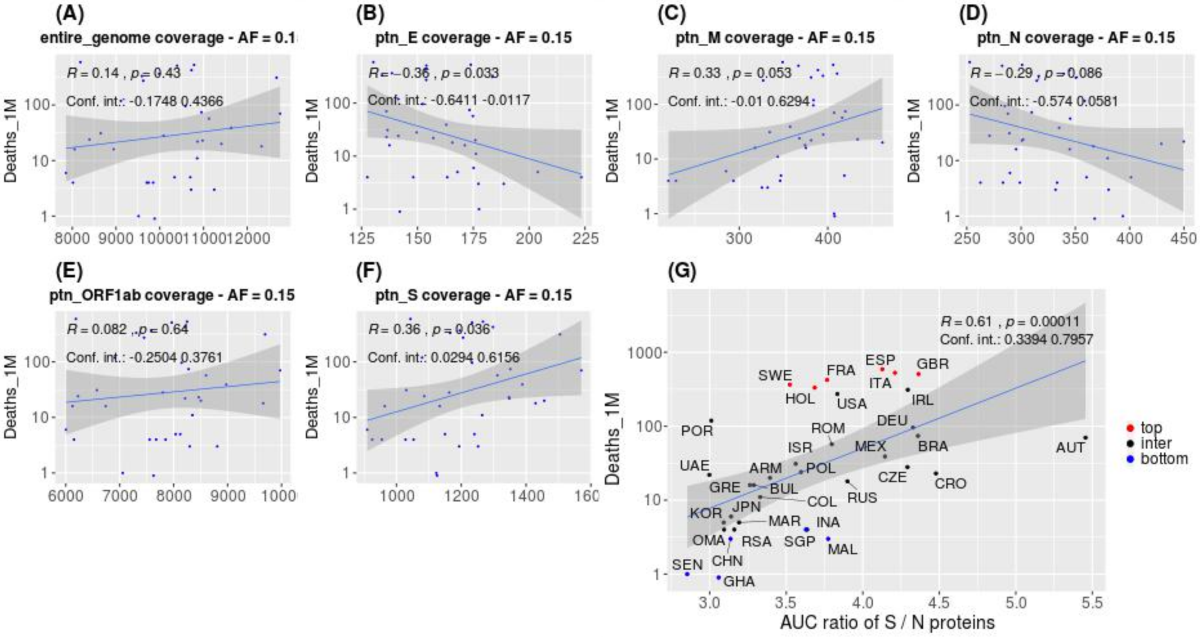

Supplementary Figure 9. Correlations between SARS-CoV-2 derived SB peptides coverage of each population and COVID-19 outcome. Spearman correlations between AUC calculated from the population coverage (cumulative AF: 0.15) considering the entire SARS-CoV-2 genome **(A)**, Envelope **(B)**, Membrane **(C)**, Nucleocapsid **(D)**, ORF1ab **(E)** or Spike protein **(F)** derived peptides and Deaths per million inhabitants. **(G)** Spearman correlation between the S/N AUC ratio and deaths per million inhabitants. Colors represent countries with a higher (red) or lower (blue) number of deaths. For this analysis, only alleles that were added to reach a cumulative AF of 0.9, starting from 0.75, for each population, were considered. The confidence interval shown was calculated using bootstrap with 1000 replacements.

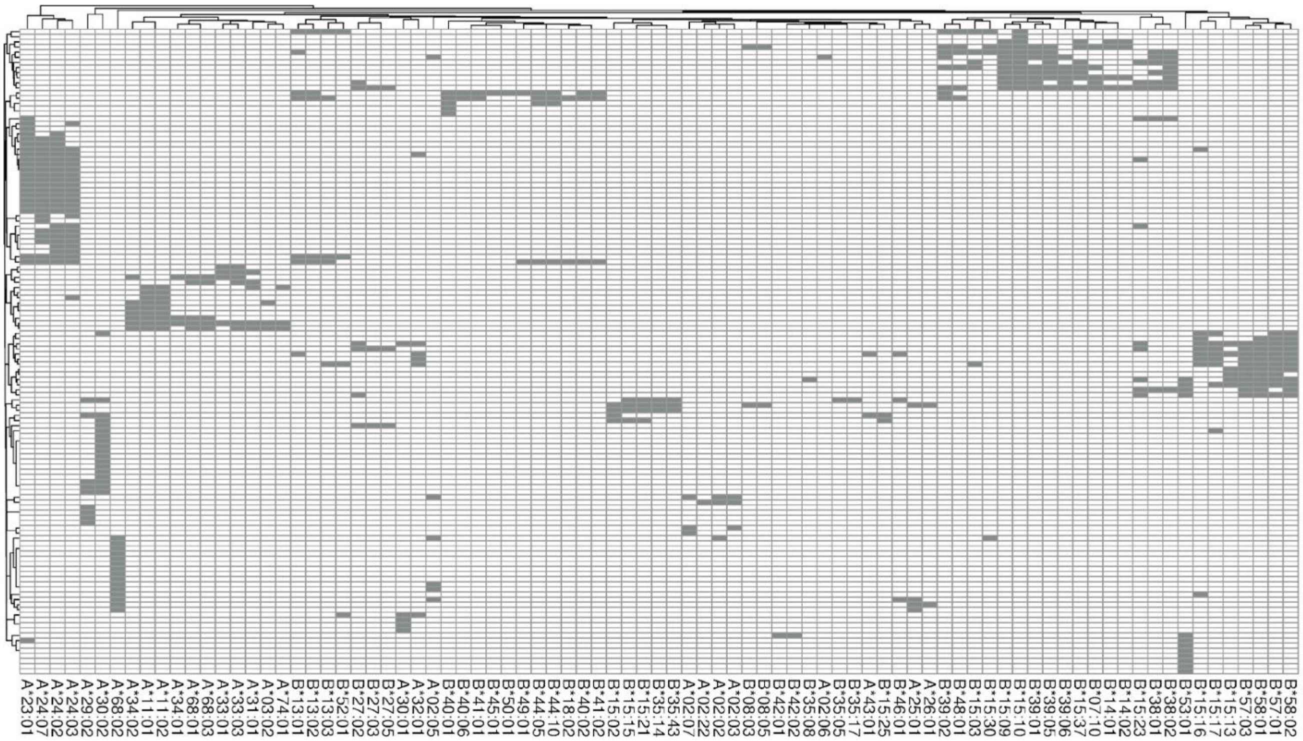

Supplementary Figure 10. Distribution of SB peptides derived from S protein potentially presented by HLA alleles with high frequency in countries of the first quartile. The complete method and 1-Pearson correlation coefficient as distance were used to cluster columns.

2.2 Supplementary Tables

Table S1. Numbers and frequencies of HLA alleles used in population coverage approaches. Values in bold represent the median for each metric.

Allele Frequency (AF)	HLA-A Cum. AF	No. of HLA-A alleles	HLA-B Cum. AF	No. of HLA-B alleles
0.75 +/- 0.04	0.721-0.781 (**0.752**)	4-14 (**7**)	0.714-0.570 (**0.739**)	6-26 (**13**)
0.90 +/- 0.02	0.888-0.910 (**0.8997**)	7-21 (**12**)	0.882-0.900 (**0.8949**)	10-41 (**22**)
0.75-0.90 (0.15)	0.118-0.1802 (**0.148**)	2-13 (**5**)	0.142-0.182 (**0.155**)	4-17 (**9**)

Table S2. Number of unique 8-11 mer Strong Binder peptides:HLA pairs for all HLA-A and B alleles. Unique peptides for each HLA gene are within parenthesis.

ORF / Protein	ORF Length (aa)	HLA-A binders	HLA-B binders
ORF1ab	7,097	5,021 (1,610)	9,545 (1,760)
ORF3a	276	260 (75)	402 (85)
ORF6	62	24 (8)	37 (11)
ORF7a	122	98 (32)	148 (28)
ORF8	122	63 (26)	81 (20)
ORF10	39	36 (11)	132 (13)
S	1,274	689 (242)	1,386 (247)
E	76	57 (17)	57 (10)
M	223	158 (50)	283 (58)
N	420	131 (52)	321 (59)

Table S3. Populational coverage calculated at IEDB considering the entire SARS-CoV-2 genome and the selected HLA-I alleles for each country.

**Country**	**Projected population coverage**	**Average number of epitope / HLA combination that is recognized**	**Minimum number of epitope / HLA combination to cover 90% of population**
ARM	99.53%	519.25	300.09
AUT	99.42%	490.81	282.21
BRA	99.58%	538.52	300.23
BUL	99.66%	528.68	317.87
CHN	99.51%	518.43	276.77
COL	99.64%	538.67	316.0
CRO	99.54%	528.74	300.55
CZE	99.56%	519.48	299.6
DEU	99.59%	535.09	302.91
ESP	99.63%	555.34	320.34
FRA	99.58%	525.54	300.37
GBR	99.6%	523.3	300.15
GHA	99.56%	500.36	299.91
GRE	99.69%	563.45	337.33
HOL	99.55%	525.38	300.4
INA	99.6%	550.96	307.23
IRL	99.46%	502.61	299.28
ISR	99.61%	547.01	300.89
ITA	99.57%	541.68	306.76
JPN	99.62%	496.96	262.92
KOR	99.52%	466.29	243.5
MAL	99.57%	552.45	325.45
MAR	99.57%	509.42	265.16
MEX	99.62%	519.22	304.47
OMA	99.65%	565.58	336.14
POL	99.59%	551.91	315.8
POR	99.54%	527.48	300.24
ROM	99.56%	540.4	324.99

RSA	99.58%	560.63	309.69
RUS	99.49%	515.27	299.29
SEN	99.53%	536.58	296.68
SGP	99.55%	525.14	312.26
SWE	99.42%	484.73	277.53
THA	99.59%	559.58	334.73
TUN	99.58%	522.79	277.93
UAE	99.55%	482.44	248.84
USA	99.67%	551.5	320.62
Average	99.57	527.61	300.68
Standard deviation	0.061	24.26	22.02
